# Cytoreductive Surgery may be beneficial for highly selected patients with Metastatic Gastrointestinal Stromal Tumors receiving Regorafenib facing Local Progression: A Case Controlled Study

**DOI:** 10.7150/jca.50324

**Published:** 2021-04-12

**Authors:** Chun-Nan Yeh, Chia-Hsiang Hu, Shang-Yu Wang, Chiao-En Wu, Jen-Shi Chen, Chun-Yi Tsai, Jun-Te Hsu, Ta-Sen Yeh

**Affiliations:** 1 GIST Team, Department of Surgery, Chang Gung Memorial Hospital, Linkou; Chang Gung University, Taiwan.; 2 GIST Team, Division of Hematology-Oncology, Department of Internal Medicine, Chang Gung Memorial Hospital, Linkou; Chang Gung University, Taiwan.

**Keywords:** cytoreductive Surgery, GIST, regorafenib, local progression

## Abstract

**Background:** Current evidence have shown surgery may provide progression-free survival (PFS) benefit for selected patients with metastatic gastrointestinal stromal tumor (GIST) who received first line imatinib and second line sunitinib. However, impact of cytoreductive surgery for GIST patients receiving third line regorafenib facing progression is not yet reported.

**Methods:** Between 2014 and 2019, 41 patients with metastatic GIST received regorafenib and 37 of them facing progression.

**Results:** 37 of 41 (90.2%) pre-treated GIST patients receiving regorafenib who experienced progression of disease after a median follow-up of 12.42 months after regorafenib use and 15 out of 37 (40.5%) patients with local progression underwent cytoreductive surgery (local progression and operation, LPOP). All the patients facing local progression (LP) were significantly younger with more exon 17 mutation than diffuse progression (DP). The complication rate for cytoreductive surgery was 33.3% (5/15). Cytoreductive surgery provided PFS prolongation of 5.52 months. Patients underwent cytoreductive surgery, compared with control group (local progression and no operation (LPNOP) and DP), may gain a significant PFS (12.91 versus 2.33 versus 5.29 months, *p* = 0.0001) and overall survival (OS) benefit (32.33 versus 5.26 versus 12.42 months, *p* = 0.004).

**Conclusions:** Cytoreductive surgery might be feasible in highly selected patients with pre-treated GIST who are being treated with regorafenib experiencing LP.

## Introduction

Since 2001, imatinib mesylate (IM) has become the first line therapy for patients with metastatic gastrointestinal stromal tumors (GISTs). We observed metastatic GIST patients experienced durable periods of disease control by IM treatment lasting from months to years, but they eventually faced disease progression with a median progression-free survival (PFS) of 37 months 1. Furthermore, cytoreductive surgery has been proposed as one of the treatment modalities to achieve long-term PFS and OS in patients with metastatic GIST on IM therapy 2-8. However, we still cannot define cytoreductive surgery combined with IM actually improves prognosis than IM therapy alone (without any surgery) in the subset of patients with stable or responsive disease on IM because there is no phase III randomized trial successful due to poor patients enrollment 9.

Sunitinib malate (SU), a multi-targeted tyrosine kinase inhibitor, has become the second-line treatment when patients who develop resistance to or are intolerant of first-line IM treatment. We ever reported the median PFS for metastatic GIST patients treated with SU is 11 months 10. Although regorafenib is approved as the third-line therapy for IM- and SU-pre-treated GIST patients currently 11, appropriate treatment for patients with progression of disease and with regorafenib treatment has not been investigated. According to Asian GIST consensus guideline, there is no evidence to recommend surgery during SU or regorafenib 12. Although we reported surgery may provide survival benefit in selected patients receiving SU facing LP 10, the impact of surgery on patient with regorafenib facing LP is still unknown. Most importantly, in Taiwan, the clinical trial is not easily accessible and even unavailable; the role of cytoreductive surgery, which may provide patients more time till newly developed therapy available, in these pre-treated GIST patients should be fully defined. We herein report the feasibility of cytoreductive surgery, PFS, and OS specifically in patients with metastatic GISTs who were taking regorafenib and experiencing LP.

## Methods

### Patient selection and preoperative management

Between 2014 and 2019, 41 metastatic GIST patients receiving regorafenib therapy in the Department of Surgery at Chang Gung Memorial Hospital (CGMH) were enrolled. All the patients were managed by a multidisciplinary GIST team composed of medical oncologists and surgical oncologists.

Pathological diagnoses of GIST were confirmed using standard hematoxylin/eosin staining and CD117 immunohistochemistry on formalin-fixed, paraffin-embedded tissues. Tissues were fixed with 10% formalin at room temperature for ≥24 h. Subsequently, PCR was performed as previously described 13 on the DNA isolated from these sections to amplify the genomic DNA sequences of KIT and PDGFRA by Professor CY Tzen at Cathay Memorial Hospital (Taipei, Taiwan). Sequences for mutations of KIT and PDGFRA were analyzed as described previously 13.

Patient data were prospectively collected and recorded in a departmental GIST database. This retrospective study was approved by the local institutional review board of CGMH. Written informed consent for the analysis of tumor-associated genetic alteration was obtained from each patient.

We prospectively collected the data from the medical record, including initial presentation (primary/metastatic), details of all operations for primary and metastatic disease, best responses to IM, SU, and regorafenib, response to regorafenib, disease extent, and extent of surgery at the time of surgery on regorafenib, post-operative complications, progression after surgery while on regorafenib, last follow-up, and, whenever applicable, death, and then retrospective analyzed all aforementioned parameters. All patients were treated with IM and SU prior to starting regorafenib. Regorafenib dose and dosing intervals were determined by the protocol under which the patient was enrolled. We used Response Evaluation Criteria in Solid Tumor group (RECIST) criteria to define the best responses to IM, SU, and regorafenib therapy as either complete response, partial response, stable disease, or progressive disease 14. All patients with LP were grouped together for analysis. LP, local progression, was defined as previous study by Raut et al in 2006 6. Under the condition of LP, most of lesions are either stable or responding to regorafenib therapy and, importantly, all lesions with progression can be resected theoretically.

### Surgery and post-operative management

Decisions to proceed with cytoreductive surgery were made jointly by the GIST team, including medical and surgical oncologists. Although the optimal interval for being off-the-drug was not yet known, regorafenib was discontinued the day before elective surgery. We performed cytoreductive surgery to remove all sites of disease in patients with LP and preserve organ function to the greatest extent possible. Once the aforementioned goals were achieved, most patients underwent further cytoreduction of as much additional disease as possible, given the constraints of the patient's overall health and the location and extent of disease. Most patients resumed their preoperative regorafenib regimen at the time of their first post-operative visit to clinic. Post-operative follow-up consisted of physical examination and computed tomography (CT) scans at 3-month intervals.

Because the goal of the analysis was descriptive, PFS and OS times were measured from several different points of treatment. PFS was measured from the date of regorafenib therapy initiation, and the date of surgery after regorafenib until the date of documented progression of residual disease, disease recurrence, or death from any cause, whichever occurred first. OS was measured from the initial date of regorafenib therapy initiation until the date of death from any cause. The Clavien-Dindo classification of surgical complications was applied 15. Surgical mortality was defined as death within one month after surgery.

### Statistics

All data are presented as percentages of patients or median with range. All numerical continuous data were compared by independent Student's *t*-tests. Categorical data were compared by Pearson's chi-squared tests or Fisher's exact tests and multiple forward stepwise logistic regression analyses when appropriate. The survival rates were calculated using the Kaplan-Meier method. Survival analyses were performed using the log-rank test, and the Cox proportional hazards model was employed for multivariate regression analyses. IBM SPSS Statistics for Windows (Version 26.0, Chicago, IL, USA) was used for statistical analyses. *P* < 0.05 was considered statistically significant.

## Results

### Initial therapy

Between April 2014 and December 2019, 41 patients who were refractory or intolerant to imatinib and sunitinib and with measurable disease based on the RECIST were enrolled. Supplementary Table 1 summarized the demographic data and initial treatment details of these 41 patients treated with regorafenib. Among this cohort (27 male and 14 female patients), all patients were treated with IM for metastatic disease for a median of 62.03 months (range, 12.48-148.73 months) prior to starting SU therapy and treated with SU for metastatic disease for a median of 16.99 months (range, 0.46-78.19 months) prior to starting regorafenib therapy, respectively.

### Clinical information of present cohort

In terms of best response, 4 out of 41 GIST patients who received regorafenib experienced partial response and 16 out 41 GIST patients who received regorafenib experienced stable response with the median time to response of 2.78 and 2.32 months, respectively. The overall disease control rate to regorafenib for the 41 GIST patients is 48.78% (Table 2). Till last follow-up time, 37 out of 41 GIST patients (90.2%) who received regorafenib experienced progression. For the 37 patients, 21 (56.8%) facing LP while 16 (43.2%) facing diffuse progression. Among them, 15 patients (40.5%) with LP received cytoreductive surgery, and the other 6 patients did not (Table 1). The median age of LP group patients is significantly younger than that of the DP group patients with more exon 17 mutation (57.14% versus 12.5%, *p* value=0.007) which was examined within 2 months of regorafenib use.

Supplementary Table 2 summarizes the demographic data and initial treatment details of these 15 metastatic GIST patients with regorafenib facing LP receiving surgery before regorafenib treatment. There were 8 males and 7 females with median age of 28 years old when they were diagnosed with GIST. Cytoreductive surgery was performed after a median of 4.88 months after regorafenib initiation. Almost all the operations were performed electively and only one patient received emergent surgery due to gastrointestinal bleeding.

### Detailed tumor location, surgical procedures and surgical results

Table 3 summarized tumor characteristics between patients with or without surgery when LP after regorafenib treatment. LP occurred most commonly as intra-abdominal tumor (10/21; 47.62%), followed by liver (7/21; 33%) and peritoneal lesion (6/21; 28.57%). Resection of intra-abdominal tumor (10/15; 66.7%) comprised the most common surgical procedures and followed by resection of the peritoneal lesion (5/15; 33.3%). Gross tumor clearance with negative evidence of disease was achieved in 26.7% (4/15) of patients with local progressive disease. While the number of lesions was smaller in surgical group, both maximal size of lesion and size summation of lesions were larger in patients without surgery. Surgical complexity score groups are lower in the non-surgical group than surgical group. This may be due to the surgical complexity scores mainly depend on the operative procedures performed based on organ involved and not consider the tumor numbers or sizes.

Five patients (5/15; 33.3%) experienced grade II to III complications, including one gastrointestinal bleeding, one surgical wound infection, one intestinal perforation, one intra-abdominal seroma formation, and one pancreatic stump leakage (Table 4). There was no post-operative death after cytoreductive operation. The length of hospital stays of these 15 patients ranged from 7 to 59 days (median: 13 days).

### Progression-free and overall survival

**Figure 1:**
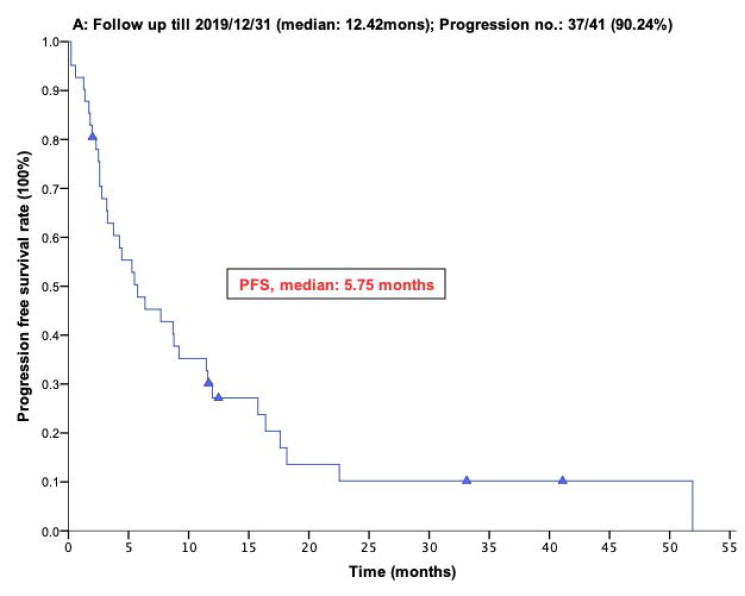

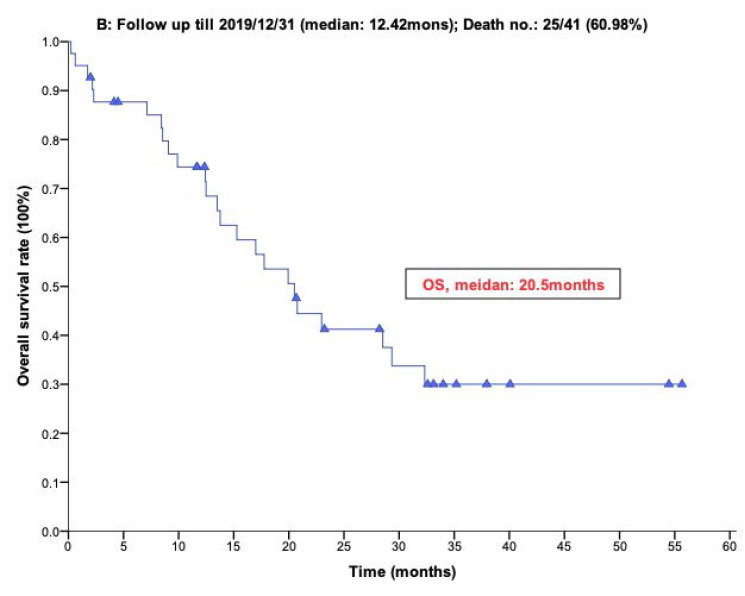
For 41 Taiwanese patients with metastatic GIST treated with Regorafenib. **(A)** Progression free survival rate; **(B)** Overall survival rate.

**Figure 2:**
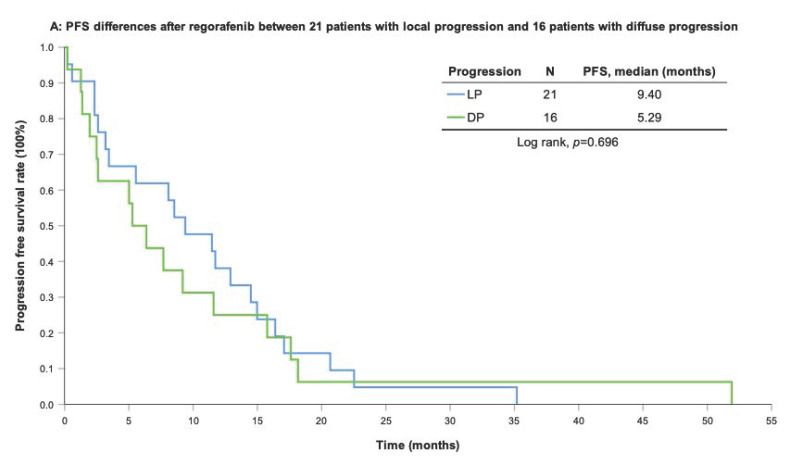

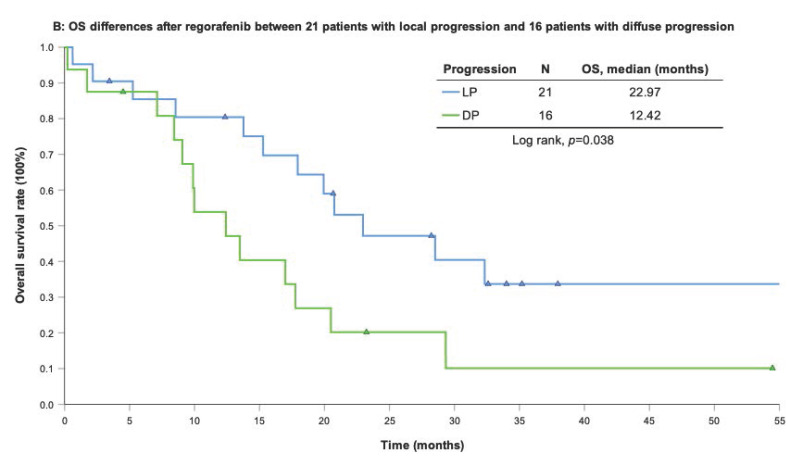
For survival rate between local progression and diffuse progression. **(A)** Progression free survival rate; **(B)** Overall survival rate.

**Figure 3:**
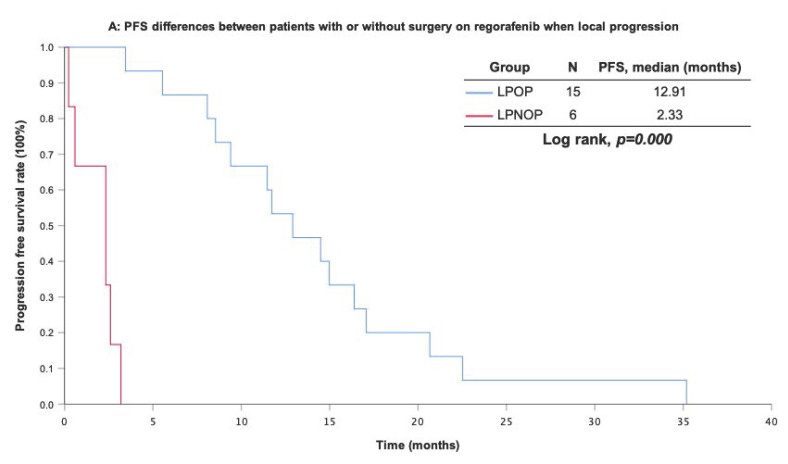

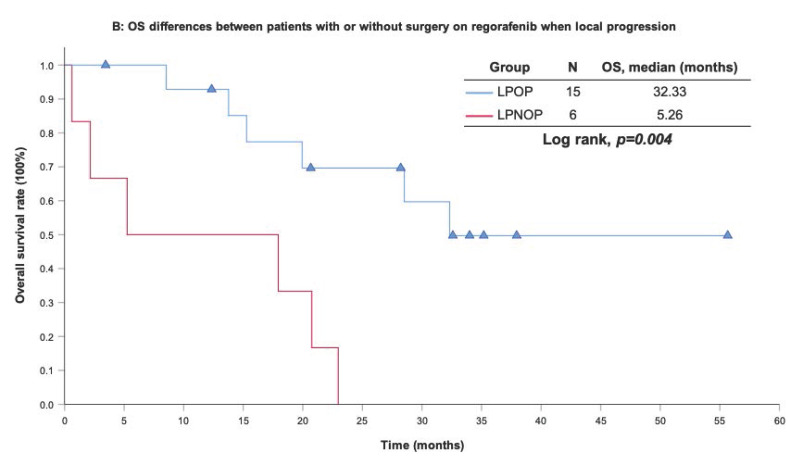
For survival rate between patients with or without surgery on regorafenib when local progression. **(A)** Progression free survival rate; **(B)** Overall survival rate.

**Figure 4:**
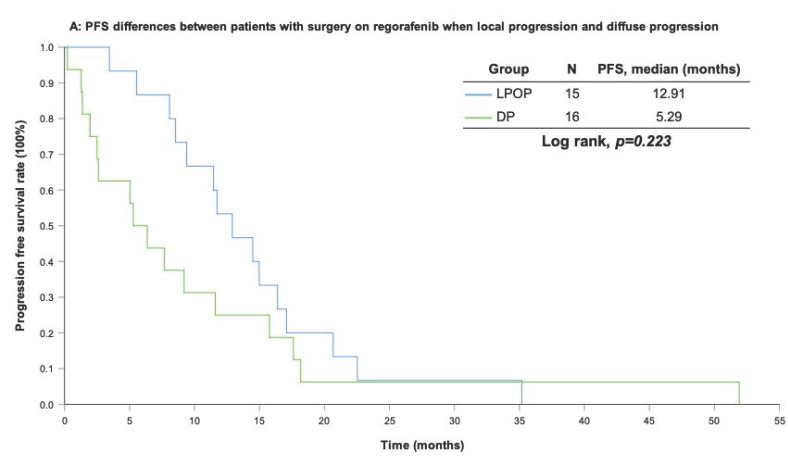

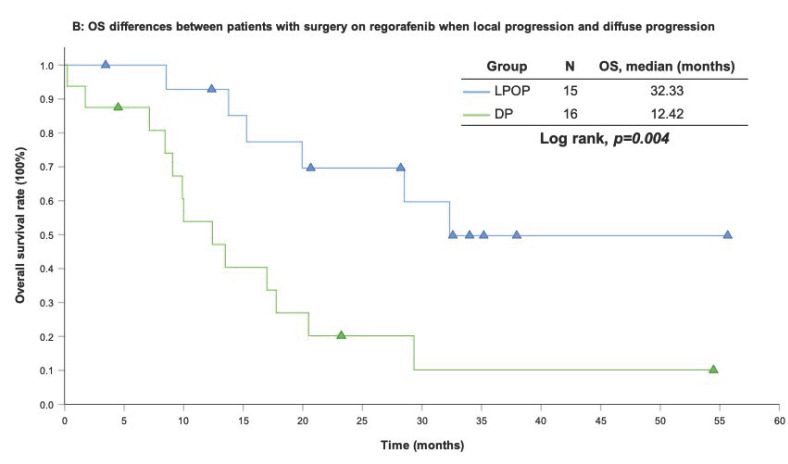
For survival rate between patients with surgery on regorafenib when local progression and diffuse progression. **(A)** Progression free survival rate; **(B)** Overall survival rate.

With a median follow-up period of regorafenib use for 12.42 months, disease progression was noted in 37 out of 41 patients (90.24%) with a median PFS and OS of 5.75 and 20.5 months, respectively (Figure 1A, 1B). The prolongation of median PFS was 5.52 months for 15 GIST patients with regorafenib therapy who experienced LP after cytoreductive surgery (Table 5). The median OS was 25.59 months after cytoreductive surgery, 32.33 months after start of regorafenib and 91.96 months after initial diagnosis of metastatic GIST receiving imatinib (Table 5). GIST patients on regorafenib with LP (N=21) may have longer PFS but not reach significance statistically and have significantly prolonged OS when compared with DP (N=16) (12.91 versus 2.59 in PFS and 32.33 versus 12.42 months in OS; *p* = 0.696 and *p* = 0.038, respectively; Figure 2A and Figure 2B). The PFS between LPOP vs. LPNOP and LPOP vs. DP were 12.91 vs. 2.33 and 12.91 vs. 5.29 months; *p* = 0.000 and 0.223, respectively (Figure 3A and Figure 4A). The OS between LPOP vs. LPNOP and LPOP vs. DP were 32.33 vs. 5.26 and 32.33 vs. 12.42 months; *p* = 0.002 and 0.004, respectively (Figure 3B and Figure 4B).

## Discussion

In Taiwan, challenges exist for patients with IM- and SU-pre-treated metastatic GIST who were treated with regorafenib and experienced objective progression because only few treatment options have been provided. In addition, there are no clinical trials for new therapy are available in Taiwan. For example, BLU-285 is reported well-tolerated and provides broad mutational coverage in pre-treated GIST patients 16. A phase III randomized study comparing BLU-285 to regorafenib as third-line therapy for GIST is planned to begin. However, we cannot participate in this trial in Taiwan. Therefore, other local ablative management, including surgery, should be still considered as a treatment option for metastatic GIST after progression and failure of regorafenib. This study mainly demonstrated the impact of surgery on progression after regorafenib use, specifically for LP.

As demonstrated in this study, regarding patients of LP treated with cytoreductive surgery, further progression of disease in a median of 5.52 months after surgery is inevitable. Our study demonstrated that surgery for selected patients of LP could significantly prolong PFS and OS after surgery to as long as 12.91 and 32.33 months, respectively, and present result demonstrated better clinical outcome of these selected group, compared with those of LP patients without cytoreductive surgery and DP patients. In addition, LP patients who undergoing cytoreductive surgery in our cohort literally had “additional PFS”, compared to regorafenib alone in previous reported study 11. Consistent with our previous study, progression of the intra-abdominal tumor is main cause of LP, although it could be bias for the accessibility for surgical intervention in the LP scenario 8. Therefore, surgery may be regarded as a bridging strategy and combined with other local ablative procedures to prolong survival of selected patients with LP and the prolonged survival could provide the patient an opportunity for treatment with the available next-generation tyrosine-kinase inhibitor or even immunotherapy.

The issue regarding surgical complication for GIST patients with targeted therapy needs attention. Raut et al have emphasized surgery is feasible in patients with metastatic GIST on SU, but incomplete resections are frequent and complication rates are high 17. Relevance of survival rates is difficult to assess given the selection bias. We still cannot define cytoreductive surgery combined with IM actually improves prognosis than IM therapy alone (without any surgery) in the subset of patients with stable or responsive disease on IM because there is no phase III randomized trial successful due to poor patients enrollment 9. This fact is even more true for 3^rd^ line therapy. Therefore, benefits of surgery should be weighed against symptoms and alternative treatments 17. Recently, they further proposed the surgical complexity score can predict morbidity, which may help in preoperative risk stratification and optimal treatment planning 18.

The surgical complication rate for LP patient in our cohort with regorafenib receiving cytoreductive surgery in this study is 33.3% (5/15). For patients with IM and SU undergoing surgery, the surgical complication rate is 13.2% and 15.3%, respectively (5/38 and 4/26, respectively) 8,10. Compared with primary GIST patients receiving surgery without targeted therapy, the surgical complication rate is significantly high. The possible mechanism responsible for the higher complication is multi-factors including dysregulation of PDGFR and MMPs and altered microarchitecture of tissue despite withdrawal of TKIs days before the operation 19. So, the surgery should be performed by experienced surgeons, especially those who are familiar with combination therapy including surgery and targeted therapy.

Although our results may support a potential impact of cytoreductive surgery on patients with GISTs who experience LP after regorafenib treatment, there were several limitations inherent to this study. First, this was a retrospective study, and although all data were collected prospectively, selective and recall bias could still not be completely prevented. Secondly, patients in our cohort underwent surgery were younger with good performance. Since there has been no prospective randomized study to demonstrate efficacy of surgery to patients with advanced GISTs who experienced LP after regorafenib treatment, we have to arrange cytoreductive surgery very carefully and prevent patients from complications. Under this general principle, we only recruited patients physically fit for this treatment. Even with this highly selective practice, postoperative complication rate was high. Therefore, selection bias is inevitable under the safety consideration. Additionally, this study was not double-blinded. Therefore, biases related to attitude of both patients and surgeons may exist. The unblinded patients may have had more motivation to receive therapy because they thought they could undergo surgery. Unblinded surgeons may have been more aggressive in facilitating therapy after the operation. Hence, the placebo effect could not be completely avoided in this study. To overcome these limitations, our results should be confirmed by further prospective randomized controlled trials that examine surgical benefits for patients with advanced GISTs who experience LP after regorafenib treatment.

In conclusion, cytoreductive surgery might be beneficial in highly selected patients with pre-treated GIST who are being treated with regorafenib experiencing LP compared to LP without operation, demonstrated by longer PFS and OS after cytoreductive surgery by experienced surgeons. This strategy may be considered as a bridging strategy for patient to further novel medication.

## Supplementary Material

Supplementary tables.

## Figures and Tables

**Table 1:** Demographic data between patients with or without surgery on regorafenib when local progression or diffuse progression (*N*=15 versus 6 versus 16)

	LPOP (N=15)	LPNOP (N=6)	*p* value (LPOP vs LPNOP)	DP (N=16)	*p* value (LP vs DP)
**Age (years), median (range)**					
Diagnosis of GIST	28 (28-63)	52 (39-57)	0.081	53 (21-82)	0.012*
Diagnosis of metastasis	35 (30-65)	52 (39-60)	0.168	57 (22-82)	0.009*
Start of Imatinib	31 (30-65)	53 (39-60)	0.080	53 (21-82)	0.015*
Start of Sunitinib	44 (36-69)	60 (41-62)	0.120	57 (23-85)	0.004*
Start of Regorafenib	45 (36-69)	62 (41-64)	0.091	61 (25-85)	0.035*
Surgery after Regorafenib	45 (36-70)	N/A		N/A	
**Gender, number (%)**					
Male	8 (53.3)	4 (66.67)	0.659	10 (62.5)	0.315
Female	7 (46.7)	2 (33.33)		6 (37.5)	
**ECOG, number (%)**					
0-1	13 (86.67)	3 (50)	0.115	9 (56.25)	0.291
2-3	2 (13.33)	3 (50)		7 (43.75)	
**Exon 17 mutation (%)**					
No	5 (33.33)	4 (66.67)	0.331	14 (87.5)	0.007*
Yes	10 (66.67)	2 (33.33)		2 (12.5)	
**Best response on Regorafenib (%)**					
PR+SD	9 (60.00)	0 (0)	0.019*	8 (50)	0.746
PD	6 (40.00)	6 (100)		8 (50)	

LPOP: local progression and operation, LPNOP: local progression and no operation, DP: diffuse progression, PR: partial response, SD: stable disease, PD: progressive disease, *p value<0.05.

**Table 2:** Antitumor response (best response) of advanced GIST treated with regorafenib (*N*=41)

Response	N (%)	Rego duration (median, mon)	TTR or TTP (median, mon)	OS (median, mon)
PR	4 (9.76)	14.65	2.78	20.50
SD	16 (39.02)	10.32	2.32	17.38
PD	20 (48.78)	3.32	2.60	13.63
N/A	1 (2.44)	2	N/A	N/A

PR: partial response; SD: stable disease; PD: progressive disease; N/A: not available; TTR: time to response; TTP: time to progression; OS: overall survival.

**Table 3:** Tumor characteristics between patients with or without surgery when local progression after regorafenib treatment (*N*=15 versus 6)

	Surgery	
	Yes (N=15)	No (N=6)
**Tumor location (case number)**		
Peritoneal	5	1
Intraabdominal tumor	10	0
Stomach	2	0
Omentum	3	0
Mesentery	3	0
Duodenum	1	0
Small bowel	2	0
Retroperitoneal tumor	2	0
Liver	3	4
Pelvis	1	0
Diaphragm	1	0
Pleural	0	1
Lung	0	1
Pericardium	0	0
Uterus	1	0
**Tumor number**		
<5	7 (46.67%)	2 (33.33%)
6-10	1 (6.67%)	0 (0%)
>10	7 (46.67%)	4 (66.67%)
**Tumor size (max, cm)**		
<5	5 (33.33%)	0 (0%)
5-10	8 (53%)	3 (50%)
>10	2 (13.33%)	3 (50%)
**Tumor size (sum, cm)**		
<10	8 (53.33%)	0 (0%)
10-20	6 (40%)	2 (33.33%)
>20	1 (6.67%)	4 (66.67%)
**Surgical complexity score groups**		
1 (low)	10 (66.67%)	6 (100%)
2 (intermediate)	5 (33.33%)	0 (0%)
3 (high)	0 (0%)	0 (0%)

**Table 4:** Surgical outcome for 15 surgeries of metastatic GIST patients during regorafenib treatment

	No.	%
Negative evidence of disease	4	26.67
Minimal residual disease	7	46.66
Bulky residual disease	4	26.67
**Complication rate (33.33%)**		
Mortality due to repeated gastrointestinal bleeding	1	6.67
Pancreatic stump leak with local abscess s/p antibiotics	1	6.67
Residual intestinal perforation s/p 2^nd^ operation	1	6.67
Surgical site infection s/p antibiotics	1	6.67
Intra-abdominal seroma, s/p pigtail insertion	1	6.67

**Table 5:** Progression and survival for 15 surgeries of metastatic GIST patients after surgery during regorafenib treatment

	No.	%
**Disease progression after surgery on Regorafenib**		
Yes	11	73.33
No	4	26.67
	***Mons.***	***95% C.I.***
**Median PFS from:**		
Diagnosis of initial metastasis	70.11	57.15-83.07
Start of Regorafenib therapy	14.49	10.96-18.02
Surgery after Regorafenib	5.52	0.00-11.87
**Median OS from:**		
Diagnosis of initial metastasis	91.96	0.00-210.19
Start of Regorafenib therapy	32.33	N/A
Surgery after Regorafenib	25.59	N/A
**Current status**	***No.***	***%***
Alive, stationary of disease	8	53.33
Alive, with disease progression	1	6.67
Dead of disease	6	40
