# Supplementary material for: Cytoreductive Surgery may be beneficial for highly selected patients with Metastatic Gastrointestinal Stromal Tumors receiving Regorafenib facing Local Progression: A Case Controlled Study
Source: J Cancer. 2021 Apr 12;12(11):3335–43. doi: 10.7150/jca.50324 (PMC8100794; doi:10.7150/jca.50324)
Supplement: Supplementary file 1 — Supplementary tables. [file jcav12p3335s1.pdf]

Supplementary Table 1. Demographic characteristics of patients with advanced GIST treated with regorafenib (*N* =41)

		Range or %
**Median age at time of (years)**		
Diagnosis of GIST	50	21–82
Diagnosis of metastasis	52	22–82
Start of imatinib	51	21–82
Start of sunitinib	55	23–85
Start of regorafenib	59	25–85
**Gender**		
Male/Female	27/14	65.85/34.15
**ECOG**		
0, 1/2, 3	27/14	65.85/34.15
**Pattern of genetic mutation***		
Exon 9	5	12.20
Exon 11	8	19.51
Exons 11 & 13	3	7.32
Exons 11 & 17	14	34.15
Exons 10 & 11 & 13	1	2.44
Exons 11& 13 & 17	2	4.88
Wild type	1	2.44
N/A	7	17.07
**Median length of imatinib treatment (months)**	62.03	12.48–148.73
**Median length of sunitinib treatment (months)**	16.99	0.46–78.19
**Primary site**		
Stomach	8	19.51
Small bowel	32	78.05
Rectum	1	2.44
**Metastatic site**		
Liver	32	78.05
Peritoneum	31	75.61
Lung	3	7.32
Others (adrenal, retroperitoneal, spine)	7	17.07
**Prior failed tyrosine kinase inhibitor**		
Imatinib	41	100
Sunitinib	41	100

ECOG: Eastern Cooperative Oncology Group; *genetic mutation represents all the cumulative exon mutation for every patient at each surgical specimen

Supplementary Table 2. Demographic data, imatinib and sunitinib treatment for 15 surgeries of metastatic GIST patients before regorafenib use		
**Median age of time of (years)**	**No.**	**Range or %**
Diagnosis of GIST	28	28-63
Diagnosis of metastasis	30	30-64
Start of Imatinib	31	30-65
Start of Sunitinib	44	36-69
Start of Regorafenib	45	36-69
Surgery after Regorafenib	45	36-70
**Gender**		
Male	8	53.3
Female	7	46.7
**ECOG**		
0-1	13	86.7
2	2	13.3
**Kit *Exon 17* mutation**	10	66.67
**No. operation prior to Imatinib therapy**		
0	1	6.66
1	7	46.67
2	7	46.67
**Median length of Imatinib (months)**	62.03	18.37-148.74
**No. operation on Imatinib**		
0	4	26.67
1	10	66.67
3	1	6.66
**Median length of Sunitinib (months)**	11.96	1.97-48.56
**No. operation on Sunitinib**		
0	9	60
1	5	33.33
2	1	6.67
**No. operation before Regorafenib**		
0	1	6.67
1	4	26.67
2	3	20
3	2	13.33
4	2	13.33
5	2	13.33
6	1	6.67

	No.	Range or %
**Regorafenib dose at operation**		
160mg	7	46.67
120mg	6	40
80mg	1	6.67
40mg	1	6.67
**Median length of Regorafenib at surgery (months)**	4.88	2.17-14.78
**Best response on Regorafenib**		
SD	9	60
PD	6	40
**Indication for surgery**		
Local progression	14	93.33
Gastrointestinal bleeding	1	6.67
**No. operation on Regorafenib**		
1	5	62.5
2	2	25
6	1	12.5
**Systemic therapy after surgery**		
Nil	1	6.67
Imatinib	1	6.67
Sunitinib	3	20
Regorafenib		
120mg	5	33.33
160mg	5	33.33
